# Genetic Variants Associated with High Susceptibility of Premature Ischemic Stroke

**DOI:** 10.1155/2023/9002021

**Published:** 2023-11-16

**Authors:** Irma Isordia-Salas, David Santiago-Germán, Rosa María Jiménez-Alvarado, Alfredo Leaños-Miranda

**Affiliations:** ^1^Thrombosis, Hemostasis and Atherogenesis Research Unit, H.G.R. No. 1 Dr. “Carlos Mac Gregor Sánchez Navarro”, Mexican Social Security Institute, Mexico City, Mexico; ^2^Health Research Division, Highly Specialized Medical Unit of Traumatology, Orthopedics and Rehabilitation “Dr. Victorio de la Fuente Narváez”, Mexican Social Security Institute, Mexico City, Mexico; ^3^Hematology Department, Highly Specialized Medical Unit “20 de Noviembre”, Institute for Social Security and Services for State Workers, Mexico City, Mexico; ^4^Medical Research Unit in Reproductive Medicine, Highly Specialized Medical Unit No. 4, Mexican Social Security Institute, Mexico City, Mexico

## Abstract

**Background:**

Several polymorphisms had been associated with an increased risk of ischemic stroke, but results are inconclusive. The aim of this study was to examine the association between AGTR1 A1166C and TSP-1 N700S polymorphisms and ischemic stroke in a young Mexican population.

**Methods:**

In a case-control study, 250 patients ≤ 45 years of age with ischemic stroke and 250 controls matched by age and gender were included. The polymorphisms were determined in all participants by polymerase chain reaction.

**Results:**

There were statistical differences in genotype distribution (*p* = 0.01) and allele frequency (*p* = 0.001) of AGTR1 A1166C polymorphism. In contrast, there was a similar genotype distribution (*p* = 0.96) and allele frequency (*p* = 0.76) of the TSP1 N700S genetic variant between groups. Hypertension (*p* = 0.03), smoking (*p* = 0.02), and family history of atherothrombotic disease (*p* = 0.04) were associated with stroke, but not diabetes (*p* = 0.30) and dyslipidemia (*p* = 0.08).

**Conclusions:**

This is the first study in Mexican population to explore several genetic variants in young patients with ischemic stroke. Our results suggest that polymorphisms in the renin-angiotensin-aldosterone system could contribute to premature hypertension, endothelial dysfunction, atherothrombosis, vasoconstriction, smooth muscle cell migration, and proliferation. In contrast, polymorphisms in the coagulation factors are not associated with ischemic stroke. Environmental factors such as diabetes and dyslipidemia could be less important in the pathogenesis of ischemic stroke at a young age. We suggest that those polymorphisms should be determined in individuals with a family history of thrombosis to avoid the stroke development. Therefore, genotype-environmental combination could determine several possible phenotypes at different moments in life.

## 1. Introduction

Ischemic stroke (IS) is the first cause of disability and the fourth cause of death worldwide. Between 5 and 8% of all IS are on individuals 45 years of age or less [1]. Several genetic variants had been associated with an increased risk of IS [1]. However, the results are still inconclusive.

Angiotensin-converting enzyme (ACE) catalyzes the conversion of inactive angiotensin I to active angiotensin II. The D/D polymorphism is present in the ACE gene and is associated with twofold increased ACE levels compared with I/I genotype, and it has been associated with IS [2]. Angiotensinogen (AGT) interacts with renin to generate angiotensin I, the precursor of angiotensin II. Angiotensin II plays a key role in the blood pressure regulation. The M235T polymorphism is localized in the AGT gene produced by the replacement of the methionine by a threonine at position 235. Individuals with the MM genotype have higher plasma AGT levels and higher blood pressure compared to individuals with the T/T genotype, which is associated with IS [3]. Another polymorphism in the AGT gene is the T174M, which results in a C to T substitution in AGT with a change in threonine by methionine at codon 207 and is associated with IS [4]. The renin-angiotensin system plays an important role in regulation of blood pressure and electrolyte homeostasis. Angiotensin II, the major biologically active product of the system, exerts its effects via two structurally distinct receptor subtypes: angiotensin II type 1 receptor (AGTR1) and angiotensin II type 2 receptor (AGT2R). An earlier study by Möllsten et al. demonstrated that AT1R A1166 polymorphism is associated with ischemic stroke [5].

Several polymorphisms have been described in the coagulation factors, such as the G20210A, present in the coagulation factor II (FII) gene, and it is associated with increased plasma levels of prothrombin and IS [6]. Another polymorphism is the G1691A localized in coagulation factor V-denominated FV Leiden. This genetic variant is responsible for the resistance of protein C activated (RPCA), and it is associated with IS [7]. The polymorphism of G1097A in the coagulation FVII is associated with a decreased plasma level of this protein [8].

TSP-1 has functions, including promoting smooth muscle cell proliferation and migration, platelet activation, and aggregation, and the N700S polymorphism is associated with increased levels of the protein and atherosclerosis; therefore, the N700S polymorphism represents a risk factor for coronary artery disease [9].

Therefore, the aim of this study was to determine the contribution of those genetic variants and environmental factors, in the pathophysiology of ischemic stroke in young Mexican individuals.

## 2. Material and Methods

### 2.1. Study Subjects

We performed a case-control study. A total of 250 consecutive unrelated patients ≤ 45 years old with a diagnosis of IS and 250 controls matched by age and gender were included. Age, gender, and history of thrombotic events were recorded. Ischemic stroke diagnosis was determined after an acute focal neurological deficit with duration > 24 h, and it was confirmed by brain computed tomography or magnetic resonance. Cardiac and carotid or vertebral artery emboli as well as cervical arterial dissection were excluded by transesophageal and transthoracic echocardiogram, cardiac magnetic resonance, magnetic resonance angiography, and carotid and vertebral Doppler ultrasound. Patients with cerebral hemorrhage diagnosis were not included. Markers of primary thrombophilia were analyzed. We performed chromogenic assays to evaluate functional levels of antithrombin, protein C, and protein S (Diagnostica Stago). We also determined lupus anticoagulant (DVV test, DVV confirm; American Diagnostic, USA), modified activated protein C resistance (normal ratio > 2.0, Coatest+APC Resistance V-S; Chromogenix, Sweden), and anticardiolipin antibodies (normal value < 10 U; Sanofi Diagnostics Pasteur, France). Individuals with one or more thrombophilia markers were excluded from this study. Patients of 45 years old or younger were included to minimize the effect of long-term environmental influences on disease etiology. In the control group, a total of 250 individuals without history of cerebrovascular disease were included. Age, sex, use of oral contraceptives, and history of thrombotic events were recorded. Other risk factors for atherothrombotic disease were registered. Informed written consent was obtained from all subjects before enrollment.

Subjects were diagnosed with hypertension, if they fulfilled the European Society Cardiology criteria or if they were treated with antihypertensive drugs. A family history of coronary disease (CAD) was defined as CAD or sudden death in a first-degree male relative, younger than 55 years of age, or a female relative younger than 65 years of age. Patients were considered smokers, if they were currently smoking or had ceased within the last 12 months. Dyslipidemia was considered if the subject had a total cholesterol level of 200 mg/dL, or if they received medication. Individuals were considered with diabetes, if they had fasting glycemia ≤ 126 mg/dL, or if they had treatment for diabetes.

Demographic and clinical data were collected during an interview performed by a physician. We included anthropometric parameters (body weight, height, and BMI) and blood pressure (BP) and biochemical measurements such as fasting plasma glucose (FPG), glycosylated hemoglobin (HbA1c), total cholesterol, and triglycerides. The I/D, M235T, T174M, AGTR1 A1166C, G20210A, G1691A, G1097A, and TSP1 N700S polymorphisms were determined in all participants by PCR-RFLP.

### 2.2. Genotyping of Polymorphisms

Genomic DNA was extracted from whole blood samples using standard methods (QIAamp DNA Blood Mini Kit, Qiagen GmbH, Hilden, Germany).

#### 2.2.1. I/D, M235T, and T174M Genotyping (Gene Polymorphisms of Angiotensin-Converting Enzyme and Angiotensinogen and Risk of Idiopathic Ischemic Stroke (https://www.sciencedirect.com/science/article/abs/pii/S037811191831223X?via%3Dihub))

The I/D polymorphism in the gene of angiotensin-converting enzyme (ACE) gene was amplified by polymerase chain reaction (PCR), using the forward primer 5′ CTG GAG ACC ACT CCC ATC CTT TCT-3′ and 5′ GAT GTGATC ACA TTC GTC AGA T-3′, as the reverse primer. The presence of 190 bp fragments represented the D allele, and the presence of 490 bp fragments represented the I allele, and they were visualized by 2% agarose gel. The PCR was performed by 30 cycles: denaturation at 94°C for 30 s, annealing segment at 60°C for 30 s, and extension at 72°C for 30 s.

The AGT-M235T and AGT-T174M polymorphisms were investigated by polymerase chain reaction (PCR) amplification of genomic DNA followed by restriction endonuclease digestion. The PCR was performed using the following primers: 5′ GATGCGCACAAGGTCCTG-3′ and 5′ CAGGGTGCTGTCCACATGGCTCGC-3′. There was an initial denaturation at 94°C, followed by 25 cycles of one minute at 94°C, one minute at 61°C, and one minute at 72°C, followed by SfaNI restriction endonuclease digestion for 4 h at 37°C. If the codon 235 is ATG (M235), SfaNI yields a 266 bp product relative to the undigested 303 bp product (T235). The AGT-T174M genotype was determined by digestion of the same 303 bp amplified product with an existing NcoI cutting site. Digested fragments were separated by electrophoresis on 3% agarose gel and yielded 211 bp + 92 bp for the C allele.

#### 2.2.2. ATR1 A1166C Genotype

The PCR protocol is 35 cycles of denaturation at 96°C for 30 s, annealing at 53°C for 30 s, and extension at 72°C for 60 s. The AT1R 1166A allele results in 58 bp and 374 bp, and the AT1R 1166 C allele results in 58 bp, 143 bp, and 231 bp fragments.

#### 2.2.3. FII, FV, and FVII Genotypes

The PCR protocol is 35 cycles of 1 min 95°C, 1 min 60°C, and 1 min 72°C, with an initial denaturation of 5 min 95°C. The PCR product was digested by Hind III prothrombin polymorphism, Mn1I for factor Leiden, and MspI for FVII polymorphism.

#### 2.2.4. TSP-1 N700S Genotype

The PCR protocol was initial denaturation at 94°C for 5 min, followed by 32 cycles of denaturation at 94°C for 40 s, alignment at 58°C for 40 s, and extension at 72°C for 30 s. The PCR product was digested by BseNi enzyme for NN 360 bp band, NS (260, 240 + 120 bp), and SS (240 + 120 bp).

## 3. Statistical Analysis

Continuous variables were expressed as mean ± standard deviation (SD) and categorical in percentages. Differences between continuous variables were determined by Student's *t*-test and chi-square test to determine differences between categorical variables. A *p* value < 0.05 was considered as statistically significant. We used the SPSS statistical software package for the statistical analysis.

## 4. Results

Baseline characteristics of patients and controls are shown in Table 1. Mean age for the group of patients was 35.6 ± 4.8, while mean age for the control group was 34.7 ± 4.2 years (*p* = 0.65). There were no differences in terms of gender (*p* = 0.65), diabetes mellitus (*p* = 30), or dyslipidemia (*p* = 0.08) between both groups. We observed significant differences in hypertension, smoking frequency, and family history of atherothrombotic disease. Patients with thrombophilic conditions and those with cerebral hemorrhage disease were excluded. We found protein S deficiency (7), protein C deficiency (1), anticardiolipin antibodies (17), lupus anticoagulant (6), acquired activated protein C resistance (4), and antithrombin deficiency (1). Only 1 patient had 2 thrombophilic conditions. All were excluded.

Table 2 shows the genotype distribution (*p* = 0.01) and allele frequency (*p* = 0.001) of AGTR1 A1166C polymorphism between patients and control groups. There was a significant difference in the genotype distribution between both groups (OR 95% CI, 1.84 (1.67-7.49) (*p* = 0.01)) and allele frequency (OR 95% CI, 1.79 (1.26-2.55) (*p* = 0.001)).

Table 3 shows the genotype distribution and allele frequency of N700S polymorphism between groups of patients and controls. We found no significant difference in genotype distribution between both groups (OR 95% CI, 0.87 (0.67-1.49) (*p* = 0.01)) and allele frequency (OR 95% CI, 0.91 (0.49-1.71) (*p* = 0.76)).

Table 4 shows the polymorphisms in candidate genes associated with premature IS in young Mexican population, with the explanation of the possible mechanism involved in the pathogenesis of the thrombotic disease.

Table 5 shows the candidate genes of proteins associated with the development of premature idiopathic IS. The polymorphisms associated with an increased risk of ischemic stroke were the AGT M235T (*p* = 0.003), the AGT T174M (*p* = 0.001), and the AGTR1 A1166C (*p* = 0.001). In contrast, ACE I/D (*p* = 0.24), FII G20210A (*p* = NS), FV G1691A (*p* = NS), FVII G10976A (*p* = 0.91), and TSP N700S (*p* = 0.76) were not associated with high susceptibility to ischemic stroke in the same group of patients.

Table 6 shows the multiple logistic regression analysis using ischemic stroke as the dependent variable. We found that AGTR1 A1166C (*p* = 0.01), AGT M235T (*p* = 0.01), AGT T174M (*p* = 0.02), hypertension (*p* = 0.03), smoking (*p* = 0.02), and family history of atherothrombotic disease (*p* = 0.04) were independently associated.

**Figure 1:**
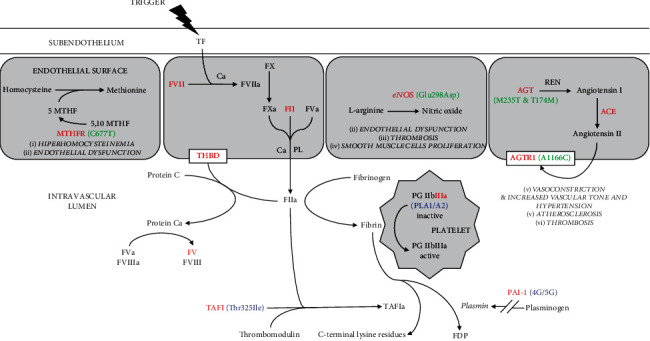
Polymorphism interaction pathways for thrombosis in young patients with ischemic stroke.

Figure 1 shows an interaction link between the single nucleotide polymorphisms associated and not associated with IS in young Mexican population.

## 5. Discussion

In our knowledge, this is the first study to analyze the association between 8 polymorphisms related with alterations in the coagulation system, endothelial dysfunction, and increased renin-angiotensin system activity with ischemic stroke (IS) in the same group of young Mexican patients less than 45 years of age.

We demonstrated that the ACE polymorphism I/D is not associated with IS [10]. Also, Pera et al. found that the D/D genotype is not associated with ischemic stroke of different etiologies in Polish population [11]. However, they found a higher percent of the D/D genotype (23.9%) compared with our results (17.6%). In contrast, Melake and Berhane [12] demonstrated that the DD genotype is associated with high susceptibility for ischemic stroke (*p* < 0.05). In that study, only 36 patients were included with a mean of age (59.4 ± 12.1). They found a higher frequency of the DD genotype (38.9%) compared to our study. Also, Goyal et al. [13] demonstrated an association in 130 patients, but only 17.5% were <40 years of age.

The RAS comprises a cascade of enzymatic reactions, which results in the production of angiotensin II (AT II) from the angiotensinogen (AGT) substrate. Renin cleaves AGT to angiotensin I (AT I), which is further converted to the bioactive octapeptide AT II through the action of ACE. In the present study, we demonstrated a significant association between AGT-M235T polymorphism and increased risk for IS (*p* = 0.001) [10]. Our findings are similar with those published by Saidi et al., who found that the AGT-M235T polymorphism was associated with an increased risk of stroke in Tunisians [14]. In a meta-analysis performed by Wang et al., including six studies with 891 ischemic stroke and 727 controls, they found significant association between AGT gene M235T polymorphism and the risk of IS as well as mortality rates on Asian population [15]. Moreover, the polymorphism T174M is also localized in the angiotensinogen (AGT) gene. In our study, the T174M was associated with IS. We found a significant difference in genotype distribution (*p* = 0.01) and allele frequency (*p* = 0.001) [10]. A previous report by Saidi et al. demonstrated a positive association between T174M polymorphism and IS in Turkish population [14].

Previous studies demonstrated that angiotensin II (AT II) contributes to atherosclerotic changes and plaque rupture by different mechanisms such as vasoconstriction, vascular smooth muscle cell growth, thrombogenesis, and decreased fibrinolysis.

It has been found that activation of human renin-angiotensin system (RAS) increases the cerebral damage in patients with ischemic stroke by AGTR1 receptor stimulation, which reduces the cerebral blood flow, and this action can produce an increase of the oxidative stress. Previous study had identified that A1166C polymorphism is localized in the AGTR1 gene [5]. We identified that A1166C polymorphism is associated with an increased risk for ischemic stroke (*p* = 0.04) in this group of young patients. Our results are in line to those published by Szolnoki et al. [16] who demonstrated that the polymorphism A1166C is associated with an increased risk of ischemic stroke in a subgroup of hypertensive patients with history of smoking (*p* < 0.001). Also, the same group found that homozygous ACE D/D genotype and at least one AT1R 1166C allele increased the risk of small-vessel ischemic stroke in a group of 308 patients with a mean of 62.3 years of age (*p* < 0.005) [17]. In a meta-analysis, Feng et al. identified that the C allele of the angiotensin II type 1 receptor A1166C gene polymorphism was associated with an increased risk for myocardial infarction in overall populations [18].

In contrast, Hulyam et al. found no association between the A1166C polymorphism and this thrombotic disease (*p* = 0.001) [19]. In a meta-analysis, Zhang et al. found that ATR1 A1166C polymorphism is not associated with susceptibility to ischemic stroke (*p* = 0.04) [20].

In the present study, the G20210A polymorphism was not present in the entire sample (patients and controls) [21]. Our results are similar with those published by Saadatnia et al., who reported no evidence of polymorphic allele A in Irani population [22]. Also, the polymorphism G1691A was not present in heterozygous GA or homozygous AA in the entire sample of patients and controls [21]. In contrast, Hamedani et al. demonstrated an association between FV Leiden and IS in patients less than 50 years of age [23]. We found no association between G10976A polymorphism and IS (*p* = 0.91) [21]. Our results are similar to those published by Maguire et al. who demonstrated that this polymorphism did not represent an increased risk for this disease. In that meta-analysis, a total of 6 studies were included, with 1537 patients and 3133 controls [24].

We found no differences related to genotype distribution or allele frequency TSP N700S polymorphism (*p* = 0.96). Also, we had previously demonstrated negative results between this polymorphism and myocardial infarction in young Mexican individuals (*p* = 0.50) [25].

We had previously demonstrated that polymorphism Glu298Asp in the endothelial nitric oxide synthase (eNOS) gene (*p* = 0.001) [26] and the C677T in the methylenetetrahydrofolate reductase (MTHFR) (*p* = 0.001) [27] were associated with an increased risk for ischemic stroke in the same group of patients. In contrast, the PIA1/A2 polymorphism of the platelet glycoprotein receptor IIIa (*p* = 0.91) [28], the 4G/5G insertion/deletion in the plasminogen activated inhibitor type 1 (PAI-1) gene (*p* = 0.40) [26], and the polymorphisms Thr325Ile (*p* = 0.20) and Ala147Thr in the thrombin-activated fibrinolysis inhibitor (TAFI) (*p* = 0.14) [29] were not associated with high risk for this atherothrombotic cerebral disease in those patients.

In our knowledge, this is the first study to analyze several polymorphisms to explain the influence of genetics in the pathophysiology of ischemic stroke in young individuals.

Hypertension (*p* = 0.002), smoking (*p* = 0.006), and family history of atherothrombotic disease (*p* = 0.007) were associated with high risk for stroke and persisted as independent risk after adjustment for other variables. In contrast with older population, diabetes mellitus (*p* = 0.30) or dyslipidemia (*p* = 0.08) was not associated. Therefore, we consider that modifiable risk factors may not have the same influence on IS in young patients compared with older individuals, and the interaction between those factors and genetic variants may be different for each group of age.

One limitation of the study was the size of the sample. However, all participants had the same ethnic background and were less than 45 years of age, groups of cases and controls were matched by age and gender, and only ischemic stroke was included. There were no patients with hemorrhagic stroke included. The 8 polymorphisms were determined in the same group of patients and controls.

## 6. Conclusions

We identified that AGTR1 A1166C, AGT M235T, and AGT T174M polymorphisms were associated with an increased risk of ischemic stroke in Mexican young individuals. Our results suggest that in young patients with IS, those polymorphisms could contribute to the premature endothelial dysfunction, increased vascular tone and hypertension, smooth muscle cell migration, and proliferation to increase the risk for this ischemic cerebrovascular disease. We observed that polymorphisms related with thrombophilic conditions, fibrinolytic activity, and increased platelet aggregability were not associated with ischemic stroke in the same group of patients: ACE I/D, FII G20210A, FV G1691A, FVII G10976A, and TSP N700S. Also, we confirmed that hypertension, smoking, and family history of atherothrombotic disease represented a risk factor for this thrombotic disease. However, diabetes, obesity, and dyslipidemia could be less important in the pathogenesis of ischemic stroke at a young age. Therefore, a specific genotype-environmental risk factor combination may determine several possible phenotypes at different moments in life.

## Figures and Tables

**Table 1:** Demographic and clinical characteristics in both study groups and controls.

	Cases	Controls	*p* value^∗^
	(*n* = 250)	(*n* = 250)	
Age, years (mean ± DE)	35.6 ± 4.8	34.7 ± 4.2	0.65
Female, *n* (%)	149 (59.6)	149 (59.6)	
Male (%)	101 (40.4)	101 (40.4)	NS
Diabetes mellitus, *n* (%)	25 (10.0)	19 (7.6)	0.30
Hypertension, *n* (%)	36 (15.2)	17 (6.8)	0.002
Smoking, *n* (%)	74 (29.6)	42 (16.8)	0.006
Dyslipidemia, *n* (%)	106 (42.4)	97 (38.8)	0.08
FH of ATD, *n* (%)	59 (23.6)	36 (14.4)	0.007

FH of ATD = family history of atherothrombotic disease; NS = nonsignificant.

**Table 2:** AGTR1 A1166C genotype distribution and allele frequency in patients with ischemic stroke and controls.

	Patients (*n* = 250)	Controls (*n* = 250)	*p* value^∗^	OR (95% CI)
Genotype				
A/A, *n* (%)	164 (65.6)	192 (76.8)	0.01^∗^	1.84 (1.67-7.49)
A/C, *n* (%)	68 (27.2)	52 (20.8)		
C/C, *n* (%)	18 (7.2)	6 (2.4)		
Dominant model				
A/A vs. A/C + C/C, *n* (%)	164 vs. 68 + 18	192 vs. 52 + 6	0.005^∗^	0.56 (0.39-0.08)
Recessive model				
C/C vs. A/C + A/A, *n* (%)	18 vs. 68+168	6 vs. 52 + 192	0.12^∗^	3.10 (1.14-8.90)
Allele frequency				
A, *n* (%)	396 (79.2)	436 (87.2)	0.001^∗^	1.79 (1.26-2.55)
C, *n* (%)	104 (20.8)	64 (12.8)		

Data presented are the number and % of patients. CI: confidence interval; odds ratio: ^∗^*X*^2^.

**Table 3:** TSP1 N700S genotype distributions and allele frequency in patients with ischemic stroke and controls.

	Patients (*n* = 250)	Controls (*n* = 250)	*p* value^∗^	OR (95% CI)
Genotype				
N/N, *n* (%)	228 (91.2%)	226 (90.4%)	0.96^∗^	0.87 (0.67-1.49)
N/S, *n* (%)	22 (4.4%)	24 (9.6%1)		
S/S, *n* (%)	0 (0.0%)	0 (0.0%)		
Dominant model				
N/N vs. N/S + S/S, *n* (%)	228 vs. 22 + 0	226 vs. 24 + 0	0.76^∗^	0.91 (0.47-1.74)
Recessive model				
S/S vs. N/S + N/N, *n* (%)	0 vs. 22 + 228	0 vs. 24 + 226	NS^∗^	NS
Allele frequency				
N, *n* (%)	478 (95.6%)	476 (95.2%)	0.76^∗^	0.91 (0.49-1.71)
S, *n* (%)	22 (4.4%)	24 (4.8%)		

Data presented are the number and % of patients. CI: confidence interval; odds ratio: ^∗^*X*^2^.

**Table 4:** Candidate genes associated to the development of premature IS.

Protein	Function	Gene	Variation type	Transcript change	Other names	Consequence
AGT	Interacts with renin to form angiotensin I	*AGT*	SNV	T803C	M235T	Higher plasma AGT levels, which result in higher blood pressure
			SNV	C620T	T174M	
ACE	Conversion of inactive angiotensin I to active angiotensin II	*ACE*	I/D of an Alu repeated sequence in an intron	N/A		Increase the serum concentration of ACE
FII	An inactive zymogen vitamin K-dependent glycoprotein, activated by FXa in the presence of phospholipids, calcium, and FVa, to form thrombin	*F2*	SNV in 3 prime UTR variant	NA	G20210A	Increase the concentration of thrombin. Thrombin converts fibrinogen to fibrin
FV	Plasma glycoprotein, when activated, acts as a cofactor for the conversion of prothrombin to thrombin by FXa	*F5*	SNV	G1601A	Leiden mutation R506G G1691A	Affects the APC cleavage site of FV, and in consequence, FVa is not properly inactivated by APC
FVII	An inactive zymogen, vitamin K-dependent glycoprotein, activated by tissue factor upon vascular injury. This complex turns on factors IX and X	*F7*	SNV	G1238A	R353Q	
AGTR1	It has vasopressor effects and regulates aldosterone secretion and controls blood pressure and volume in the cardiovascular system	*AGTR1*	SNV	A1166C	High blood pressure	
TSP-1	TSP-1 promote smooth muscle cell proliferation and migration to promote platelet activation and aggregation	*TSP-1*	SNV	N700S		Increasing smooth muscle cell proliferation and migration and platelet activation and aggregation and atherosclerosis

AGT: angiotensinogen; ACE: angiotensin-converting enzyme; FII: coagulation factor II; FV: coagulation factor V or Leiden; FVII: coagulation factor VII; FXa: activated coagulation factor X; FvW: von Willebrand factor; SNV: single nucleotide variant; I/D: insertion/deletion; APC: activated protein C; TSP: thrombospondin; AGTR1: angiotensin II type 1 receptor.

**Table 5:** Genotype distribution and allele frequencies of polymorphisms associated with ischemic stroke in young Mexican population.

Gene	Genotype frequencies						*p* value	Allele frequencies				*p* value
	Case			Control				Case		Control		
*AGT*	M235T							M235T				
	T/T	C/T	C/C	T/T	C/T	C/C		T	C	T	C	
*n* = 250	148 (59.2)	74 (29.6)	28 (11.2)	170 (68.0)	68 (27.2)	12 (4.8)	0.001	370 (74.0)	130 (26.0)	408 (81.6)	92 (18.4)	0.003
	T174M							T174M				
	C/C	C/T	T/T	C/C	C/T	T/T		C	T	C	T	
*n* = 250	186 (74.4)	60 (24.0)	4 (1.6)	208 (83.2)	41 (16.4)	1 (0.4)	0.01	432 (86.4)	68 (13.6)	457 (91.4)	43 (8.6)	0.001
*ACE*	I/D							I/D				
	I/I	I/D	D/D	I/I	I/D	D/D		I	D	I	D	
*n* = 250	88 (35.2)	118 (47.2)	44 (17.6)	98 (39.2)	116 (46.4)	36 (14.4)	0.25	294 (59.0)	206 (41)	312 (62.4)	188 (37.6)	0.24
*F2*	G20210A							G20210A				
	G/G	G/A	A/A	G/G	G/A	A/A		G	A	G	A	
*n* = 250	250 (100)	0 (0)	0 (0)	250 (100)	0 (0)	0 (0)	NS	250 (100)	0 (0)	250 (100)	0 (0)	NS
*F5*	G1691A							G1691A				
	G/G	G/A	A/A	G/G	G/A	A/A		G	A	G	A	
*n* = 250	250 (100)	0 (0)	0 (0)	250 (100)	0 (0)	0 (0)	NS	250 (100)	0 (0)	250 (100)	0 (0)	NS
*F7*	G10976A							G10976A				
	G/G	G/A	A/A	G/G	G/A	A/A		G	A	G	A	
*n* = 250	204 (81.6)	45 (18.0)	1 (0.4)	206 (82.4)	40 (16.0)	4 (1.6)	0.91	453 (90.6)	47 (9.4)	452 (90.4)	48 (9.6)	0.91
*AGTR1*	A1166C							A1166C				
	A/A	A/C	C/C	A/A	A/C	C/C		A	C	A	C	
*n* = 250	164 (65.6)	68 (27.2)	18 (7.2)	192 (76.8)	52 (20.8)	6 (2.4)	0.01	396 (79.2)	104 (20.8)	436 (87.2)	64 (12.8)	0.001
*TSP*	N700S							N700S				
	N/N	N/S	S/S	N/N	N/S	S/S		N	S	N	S	
*n* = 250	228 (91.2)	22 (8.8)	0 (0.0)	226 (90.4)	24 (9.6)	0 (0.0)	0.96	478 (95.2)	22 (8.8)	476 (95.2)	24 (4.8)	0.76

MTHFR: methylenetetrahydrofolate reductase; eNOS: endothelial nitric oxide synthase; AGT: angiotensinogen; ACE: angiotensin-converting enzyme; F2: gene of coagulation factor II; F5: gene of coagulation factor V or Leiden; F7: gene of coagulation factor VII; PAI-1gene of the plasminogen activator inhibitor type 1; CPB2: gene of thrombin activatable fibrinolysis inhibitor; OR: odds ratios; CI: confidence interval.

**Table 6:** Multiple logistic regression analysis using ischemic stroke as the dependent variable.

Risk factor	Adjusted OR (95% CI)	*p* value^∗^
AGTR 1 A1166C	1.24 (1.7-2.41)	0.01
AGT M235T	2.37 (1.4-2.61)	0.01
AGT T174M	1.06 (1.0-2.32)	0.02
Hypertension	2.65 (1.04 -4.27)	0.03
Smoking	1.67 (1.2-2.43)	0.02
Family history of ATD	1.74 (1.1-3.64)	0.04

## Data Availability

Access to data is restricted because of legal and ethical concerns and patient privacy.

## Additional Points

*Highlights*. (i) The AGTR A1166C polymorphism was associated with an increased risk for ischemic stroke (IS) in young Mexican individuals (*p* = 0.01). In contrast, there was no positive association between the TSP-1 N700S genetic variant and this thrombotic disease (*p* = 0.96). (ii) Moreover, we identified that AGT M235T (*p* = 0.04) and AGT T174M (*p* = 0.04) polymorphisms were associated with high risk for this cerebrovascular disease in this group of patients. Our results suggest that in young individuals with IS, those polymorphisms could contribute with premature increased vascular tone and hypertension, smooth muscle cell migration, and proliferation to increase the risk for this ischemic disease. (iii) Also, we observed that polymorphisms related with thrombophilic conditions were not associated with ischemic stroke in the same group of patients: I/D, G20210A, G1691A, and G10976A. (iv) Moreover, we confirmed that hypertension, smoking, and family history of atherothrombotic disease represented a risk factor for ischemic stroke. Meanwhile, diabetes and dyslipidemia could be less important in the pathogenesis of ischemic stroke at a young age. (v) In our knowledge, this is the first study that analyzed the genetic bases for ischemic stroke in young individuals in Mexican population. The influence of each polymorphism for ischemic stroke may not be the same for every race, and it is desirable to establish the contribution of each genetic variant to avoid the development of this thrombotic disease at an earlier stage of life.
